# Case Report: Novel rare mutation c.6353C > G in the *ABCA12* gene causing harlequin ichthyosis identified by whole exome sequencing

**DOI:** 10.3389/fped.2023.1128716

**Published:** 2023-02-15

**Authors:** Van Khanh Tran, Quang Minh Diep, Qiu Zilong, Le Thi Phuong, Hai Anh Tran, Nguyen Van Tung, Nguyen Thi Kim Lien, Nguyen Thi Xuan, Le Thi Ha, Thanh Van Ta, Thinh Huy Tran, Nguyen Huy Hoang

**Affiliations:** ^1^ Department of Molecular Pathology Faculty of Medical Technology and Center for Gene and Protein Research, Hanoi Medical University, Hanoi, Vietnam; ^2^ Assisted Reproductive Technology Center, Quang Ninh Hospital for Obstetric and Pediatric, Quang ninh, Vietnam; ^3^ BGI Genomics, BGI-Shenzhen, Shenzhen, China; ^4^ Institute of Genome Research, Vietnam Academy of Science and Technology, Hanoi, Vietnam; ^5^ Graduate University of Science and Technology, Vietnam Academy of Science and Technology, Hanoi, Vietnam; ^6^ Neonatal Care Center, Vietnam National Hospital of Pediatrics, Hanoi, Vietnam

**Keywords:** ABCA12, harlequin ichthyosis (HI), mutation, Vietnamese patient, whole exome sequencing (WES)

## Abstract

**Background:**

Harlequin ichthyosis (HI) is a severe rare genetic disease that mainly affects the skin. Neonates with this disease are born with thick skin and large diamond-shaped plates covering most of their bodies. Affected neonates lose the ability to control dehydration and regulate temperature and are more susceptible to infections. They also face respiratory failure and feeding problems. These clinical symptoms are factors associated with high mortality rates of neonates with HI. Until now, there are still no effective treatments for HI patients and most patients die in the newborn period. Mutation in the *ABCA12* gene, which encodes an adenosine triphosphate-binding cassette (ABC) transporter, has been demonstrated as the major cause of HI.

**Case presentation:**

In this study, we report the case who is one infant that was born prematurely at 32 gestational weeks with the whole body covered with thick plate-like scales of skin. The infant was severely infected with mild edema, multiple cracked skins full of the body, yellow discharge, and necrosis of fingers and toes. The infant was suspected to be affected by HI. Whole exome sequencing (WES) was performed as a tool for detecting the novel mutation in one prematurely born Vietnam infant with HI phenotype. And after that, the mutation was confirmed by the Sanger sequencing method in the patient and the members of his family. In this case, one novel mutation c.6353C > G (*p*.S2118X, Hom) in the *ABCA12* gene, was detected in the patient. The mutation has not been reported in any HI patients previously. This mutation was also found in a heterozygous state in the members of the patient's family, including his parents, an older brother, and an older sister who are no symptoms.

**Conclusions:**

In this study, we identified a novel mutation in a Vietnamese patient with HI by whole exome sequencing. The results for the patient and the members of his family will be helpful in understanding the etiology of the disease, diagnosing carriers, assisting in genetic counseling, and emphasizing the need for DNA-based prenatal screening for families with a history of the disease.

## Introduction

Harlequin ichthyosis (HI) is an extremely severe subtype of congenital ichthyosis with characteristic clinical features at birth including thick skin in large patches over the entire body, resulting in the skin surface hardening into large scales and flattened ears (1–3). The disease is the form with the highest mortality rate among ichthyosis and has an incidence of 1 per 300,000 live births (4,5). Ichthyoses are a group of diseases characterized by thick white, brown, or dark brown patches on the skin that cover the entire body. The thick stratum corneum that covers is shed, leaving red and scaly skin that causes severe damage to the protective layer of the skin and leads to increased transepidermal water loss, hindering thermoregulation, and increasing the risk of secondary infection (6,7). HI is an autosomal recessive genetic disease that severely affects the quality of life in patients, although other organs are not affected.

In 2005, the mutation in the *ABCA12* gene (OMIM 607800) was identified as the cause of HI (1, 8). The *ABCA12* gene (2q34) is 207 kb long and consists of fifty-three exons and two very long introns at its beginning (26.5 kb and 47.3 kb, respectively). The *ABCA12* gene codes a 2,595 amino acid protein, a member of the adenosine triphosphate (ATP)-binding cassette transporter family (1, 8–10). ABCA12 plays the role of binding and hydrolyzing ATP to transport various molecules across membranes or into vesicles in the cells (11). ABCA12 protein has a structure consisting of two transmembrane domains and two cytoplasmic domains which act as the ATP-binding cassette and are usually expressed in the cells such as the skin, brain, testis, and stomach of fetal (9). The cytoplasmic domain of the ABCA12 protein consists of 2 highly conserved motifs (Walker A and Walker B) that serve as transporter domains. The transmembrane domain consists of 6 hydrophobic transmembrane helices. In skin cells, ABCA12 is able to transport lipids such as glucosylceramide (GlcCer) through lamellar granules (LGs) which are then released onto the apical surface of granular keratinocytes to form lipid lamellae in the stratified layer (1, 12–14). ABCA12 is present in the membrane of the Golgi network and stratum corneum granules of the upper epidermis, the role mainly in the squamous and top granular cells that accumulate lipids necessary for the formation of the skin barrier (14).

When ABCA12 is mutated, lipids and proteases are not secreted into the interstitial space between the stratum corneum cells. In all areas of the body except the tongue, lipid droplets and abnormal vacuoles are clearly visible in the incompletely keratinized cells and the vacuoles contain dense granules or multilayered structures forming the white dermal layer (1). Deficiency in interstitial lipid delivery results in hyperkeratinization and epidermal inflammation and the permeability of the epidermal barrier is severely impaired leading to increased transepidermal water loss, a characteristic feature of HI (6, 7). Abnormal skin can affect the shape of the eyelids, nose, and ears and limit movement of the arms, legs, and chest leading to breathing difficulties. As a result, neonates are respiratory failure, infections, and feeding problems, which are factors that lead to a poor prognosis for HI neonates (8, 15). In addition, neonates with HI are often severely dehydrated and infected in the first few weeks after birth and are unlikely to survive.

In 2009, based on the latest knowledge of the pathogenesis of the disease, The Ichthyosis Consensus Conference classified ichthyosis syndrome into three major subtypes: Harlequin ichthyosis (HI; OMIM 242500); Congenital ichthyosiform erythroderma (CIE; OMIM 242100); and Lamellar ichthyosis (LI; OMIM 242300, 604777, 601277, 606545) (16,17). Of these subtypes, HI is considered the most severe form (8, 18). Only 83% of HI neonates who were treated with systemic retinoids survived, while the long-term survival rate was only 24% for those not receiving oral retinoids (3). HI can be differentially diagnosed because of its severe characteristic clinical features at birth, but it can also sometimes be confused with other subtypes, such as LI, which also have similar symptoms in different degrees. For a definite diagnosis, it is important to recognize a dense accumulation of lipid droplets in the granulosa cells and stratified stratum corneum cells of affected neonates' skin (19) and identify mutations in the related gene.

Until now, 124 mutations in the *ABCA12* gene have been published in the Human Gene Mutation Database (HGDM) consisting of 71 missense, 15 splicings, 23 small inserts, 6 small delete, and 7 cross-insert duplication mutations. Out of which 73 mutations were identified in HI patients. Recent studies have shown that most mutations in HI patients are truncation mutations, including nonsense mutations, frame-shift mutations (insertion/deletion mutations), and splice-site mutations (20). Most detected truncation or deletion mutations in HI patients are thought to result in the disruption of important nucleotide-binding fold domains and/or transmembrane domains, leading to a deficit of severe function of the ABCA12 protein. To date, all reported HI patients have at least one deletion and truncation mutation in the conserved region that causes severe loss of the ABCA12 function, except in one family that carries a homozygous missense mutation in the highly conserved region. In contrast, most mutations detected in LI and CIE patients are missense mutations and affect mildly ABCA12 function (20).

In this study, by WES sequencing, we have identified a homozygous mutation (c.6353C > G, *p*.S2118X) in the *ABCA12* gene in the patient. This heterozygous mutation was also detected in the members of his family. The results show that the identification of genetic mutations as the cause of HI is of great help to the DNA-based prenatal diagnosis of HI in early pregnancy with low risk in order to minimize the number of neonates with the disease.

## Case presentation

### Clinical presentation

**Figure 1:**
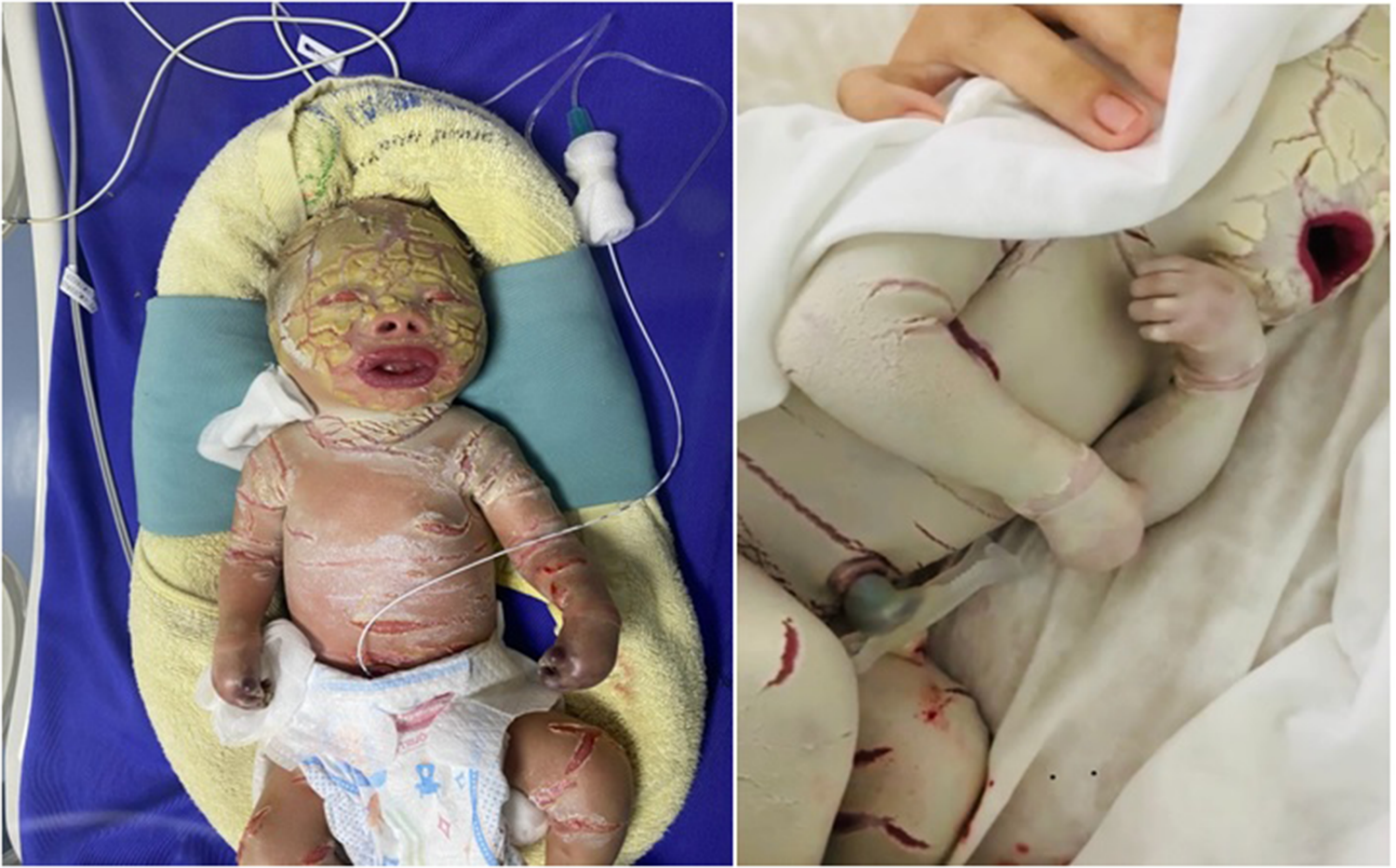
Image of patient with characteristic clinical manifestations of the HI disease. The patient's body is covered with a thick layer of skin and deep fissures. Patient also has abnormal eyes and ears; flatter lipids and nose; necrotizing fingers and toes after birth.

The case is an infant born prematurely at 32 gestational weeks in the Quang Ninh Obstetrics and Pediatrics Hospital with a birth weight of 2.2 kg and the whole body was covered with thick plate-like scales and separated by dark red fissures of the skin. His upper and lower eyelids are retractive, swollen lips open, and the nose and ears are flattened. The infant cries immediately after birth but restricted chest movement was observed, causing struggling to breathe. After 5 days, the infant was transferred to the Vietnam National Hospital of Pediatrics with severe infection, mild edema, skin with many cracks, yellow discharge, and necrosis of fingers and toes (Figure 1). The paraclinical indicators showed that there is a reduced WBC (white blood cells) count (4.69 × 10^9^/L, normal range: 7.7–14 × 10^9^/L), severely decreased platelet count (8 × 10^9^/L, normal range: 140–440 × 10^9^/L), total protein reduction (29.2 g/L, normal range: 41–63 g/L), albumin reduction (18.9 g/L, normal range: 31–43 g/L), and increased quantitative CRP level (C-reactive protein level) (149.4 mg/l, normal range: 1.6 mg/L). The infant was also identified with *Pseudomonas aeruginosa* infection. Based on the clinical phenotype, the infant was suspected to be affected by HI. He was fed with breast milk and skin hygiene and was given half of a platelet unit. After 15 h of entering the hospital, the child had difficulty breathing and died.

**Figure 2:**
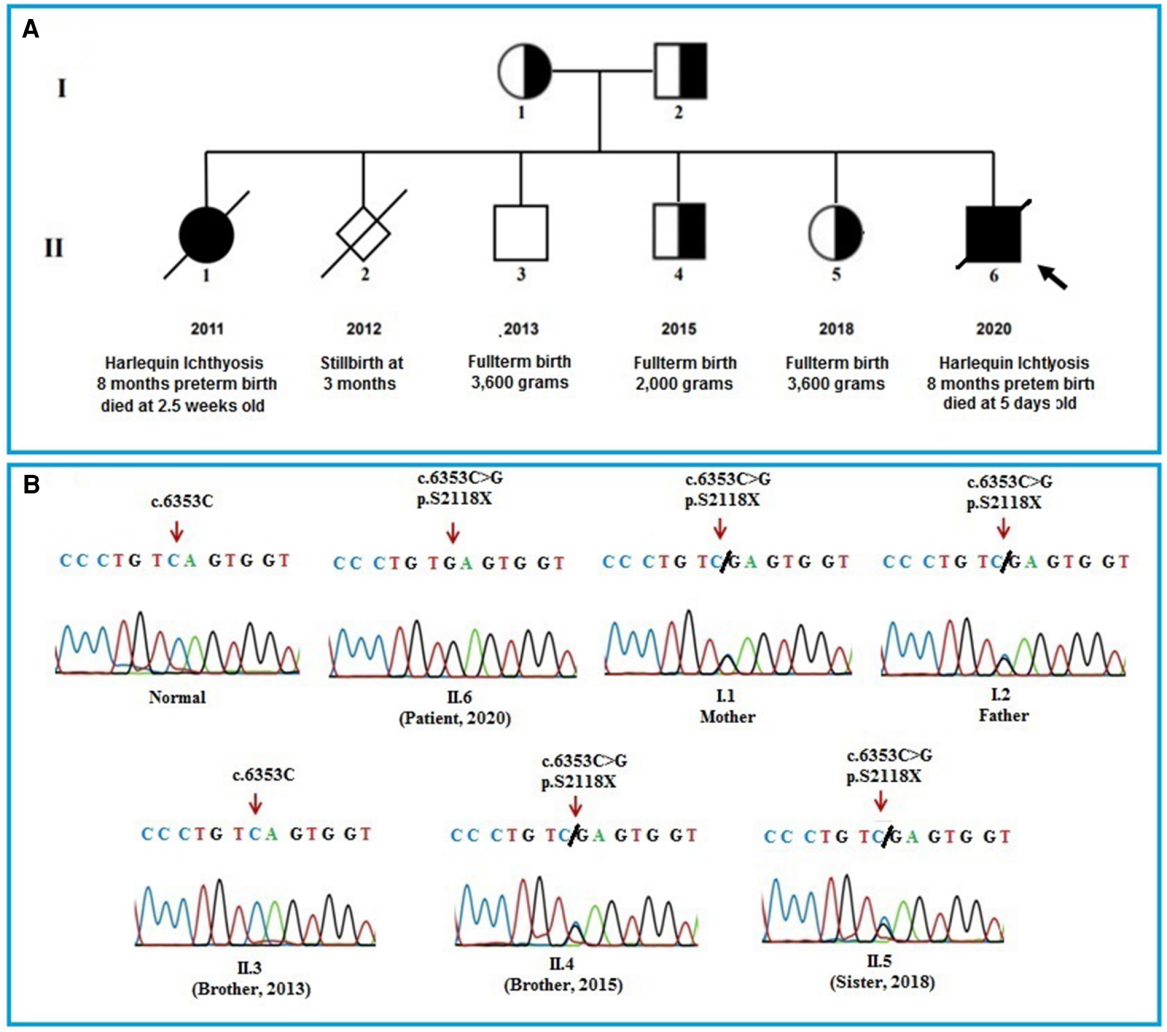
(**A**): pedigree of the patient's family. The pedigree of the patient's family including parents, a sister who died of HI disease, a stillbirth boy at 12 weeks of prenancy, a healthy brother, a brother and a sister who carrying the mutation and the patient. (**B**): The Sanger sequencing results of the members in the patient's family. The results show that the patient's parents, an older brother, and an older sister carry a heterozygous mutation (c.6353C > G) in the *ABCA12* gene. The patient bear the homozygous mutation (c.6353C > G) and the other healthy brother who was born in 2013 does not have the mutation.

The infant had a first sister, who was born 8 months premature and died at 2.5 weeks old with HI symptoms, and a stillborn brother at 12 weeks gestation. He also has two brothers and a sister with full-term healthy (Figure 2A). The patient had collected blood sample for whole exome sequencing (WES) to find the cause of the disease.

### Molecular investigation

Genomic DNA was isolated from peripheral blood samples (including of the patient and the members of his family) using a Qiagen DNA blood mini kit (QIAGEN, Hilden, German) following manufacturer guidelines. The DNA concentration was determined using a Thermo Scientific NanoDrop spectrophotometer (Waltham, MA, United States). The library was prepared with SureSelect V7-Post kit (Agilent Technology, CA, United States) following manufacturer guidelines. Whole exome sequencing (WES) was performed on Illumina NovaSeq machine (Illumina, CA, United States) in Beijing Genomics Institute. The sequencing data has the average throughput depth of target regions of 154 × and the mean depth of target regions of 49.8X. The paired-end reads were mapped to the reference human genome GRCh38 using BWA0.7.17 (21). Picard tool (http://broadinstitute.gith-ub.io/picard/) was used to process post-alignment data including creating indexes, marking, removed repeated reads on the alignment bam file. Variants calling were performed by HaplotypeCaller in the GATK package version 4.1 (22).

The gene fragments carrying nucleotide changes were amplified and analyzed by direct Sanger sequencing. Oligonucleotide primers were designed by Primer 3.0 after that were synthesized and purchased from PhusaBiochem Company (Can Tho, Vietnam) (Forward primer: ABCA12–43F 5′ TGCCTCAGCCTCCTAAAGTG 3′, Reverse primer: ABCA12–43R 5′ GATGAGGCCCAAAAAGAATTT 3′). PCR amplification of exon 40th of the *ABCA12* gene of the patient and the members of his family was carried out on an Eppendorf Mastercycler EP gradient machine (United States Scientific, Inc). DNA sequencing was sequenced in both directions, initiated from the forward and the reverse primers which had been used in the initial PCR reaction. PCR products were purified with the Qiagen PCR Purification kit (QIAGEN, Hilden, Germany) and sequenced on ABI PRISM 3,500 Genetic Analyzer machine (Thermo Fisher Scientific Inc., United States). The sequencing data were analyzed using BioEdit 7.2.5 software.

The results of WES sequencing showed that one novel homozygous mutation, c.6353C > G (*p*.S2118X) in the *ABCA12* gene, was identified in the patient. The mutation c.6353C > G (*p*.S2118X) has not been reported yet in previous studies and in the databases (1,000 Genome, gnomAD, NCBI, HGMD, UCSC, ExAc, and NHLBI). This mutation has been submitted in dsSNP under accession number rs1553520447 and Gene Dx under accession number SCV000710591.2, as well as identified as a pathogenic variant in the ClinVar database (https://www.ncbi.nlm.nih.gov/clinvar) (accession number RCV000598766.1). The Sanger sequencing method was used to validate the results in the patient and the patient's family members (Figure 2B). Both of the parents, an older brother, and an older sister of the patient carried the mutation c.6353C > G (*p*.S2118X) in heterozygous status in the *ABCA12* gene. Members of the patient's family who carry the mutation in the heterozygous state do not show symptoms of the disease. This result suggests that the homozygous mutation is the cause of the disease in the patient.

## Discussion

Harlequin ichthyosis is a rare dermatological disease characterized by a defect in the formation of the intercellular lipid layers, leading to loss of barrier function, thereby resulting in hyperkeratosis (12). In the past, affected neonates typically died within 2 days of birth from feeding problems, severe dehydration, bacterial infections, respiratory distress, and/or difficulty breathing; however, recently survivors have been reported due to neonatal intensive care and benefits of oral retinoids (23).

The most obvious clinical presentation in HI patients is an increase in the stratum corneum with large, thick, yellowish brown, and very sticky patches with dark red cracks extending to the dermis (3, 24). Facial organs, such as eyes, ears, noses, and mouth appear to be immature. Therefore, patients with HI have to deal with difficulties in eating, breathing, respiratory failure, dehydration, temperature regulation, and infections (20, 25). Patients with HI are also at high risk for hypothermia/hyperthermia, malnutrition, hypernatremia, and seizure (8). Most infants die within a few days of birth is associated with premature birth and neonatal complications such as pneumonia, sepsis, and electrolyte imbalance (26). *Pseudomonas aeruginosa* infection was found to explain the increased CRP level in infants. Pseudomonas is an important cause of fatal lung infections, especially in the neonatal intensive care unit (NICU), likely due to the neonates' underdeveloped immune systems (27). Some HI patients have been reported dead from Pseudomonas infection (28). The breathing difficulty due to severe Pseudomonas infection and the limited movement caused by the hard skin-like plating can be considered the main causes of infant mortality.

All such clinical features were observed in our patient's case. The patient was born prematurely at 32 weeks gestation, with the whole body covered by thick plate-like scales of skin, the organs such as ears, eyes, and mouth are not fully developed. After 5 days, the infant had a severe infection, mild edema, skin with many cracks, yellow discharge, and necrosis of fingers and toes. The infant was also identified with *Pseudomonas aeruginosa* infection and died due to difficulty breathing.

The *ABCA12* gene encoding for ATP-binding cassette transporter A12 is known as the pathogenic gene of HI (1, 13, 29, 30). Harlequin ichthyosis is the most severe ichthyosis subtype (8). Of all the identified mutations, most of them were truncated, deleted, or spliced mutations at the highly conserved C-terminal of the ABCA12 protein. These mutations severely affect important binding domains and/or transmembrane domains leading to a complete loss of the ABCA12 protein's transport function (8). These mutations can cause the loss of important function regions and are predicted to cause the loss of normal protein function in the truncation of the ABCA12 protein. The frequency of this mutation in the normal population is very low. In 2010, a literature review was performed in 66 unrelated families, including 48 HI, 10 LI, and 8 CIE families showing that 56 *ABCA12* mutations were described (http://www.derm-hokudai.jp/ABCA12/) (29). Of the 56 mutations, 36% are nonsense, 25% are missense, 20% comprise small deletions, 11% are splice sites, 5% are large deletions and 4% are insertion mutations. There are no obvious mutational hotspots in the *ABCA12* gene, although mutations associated with the LI phenotype are clustered in the region of the first ATP binding cassette (10).

**Figure 3:**
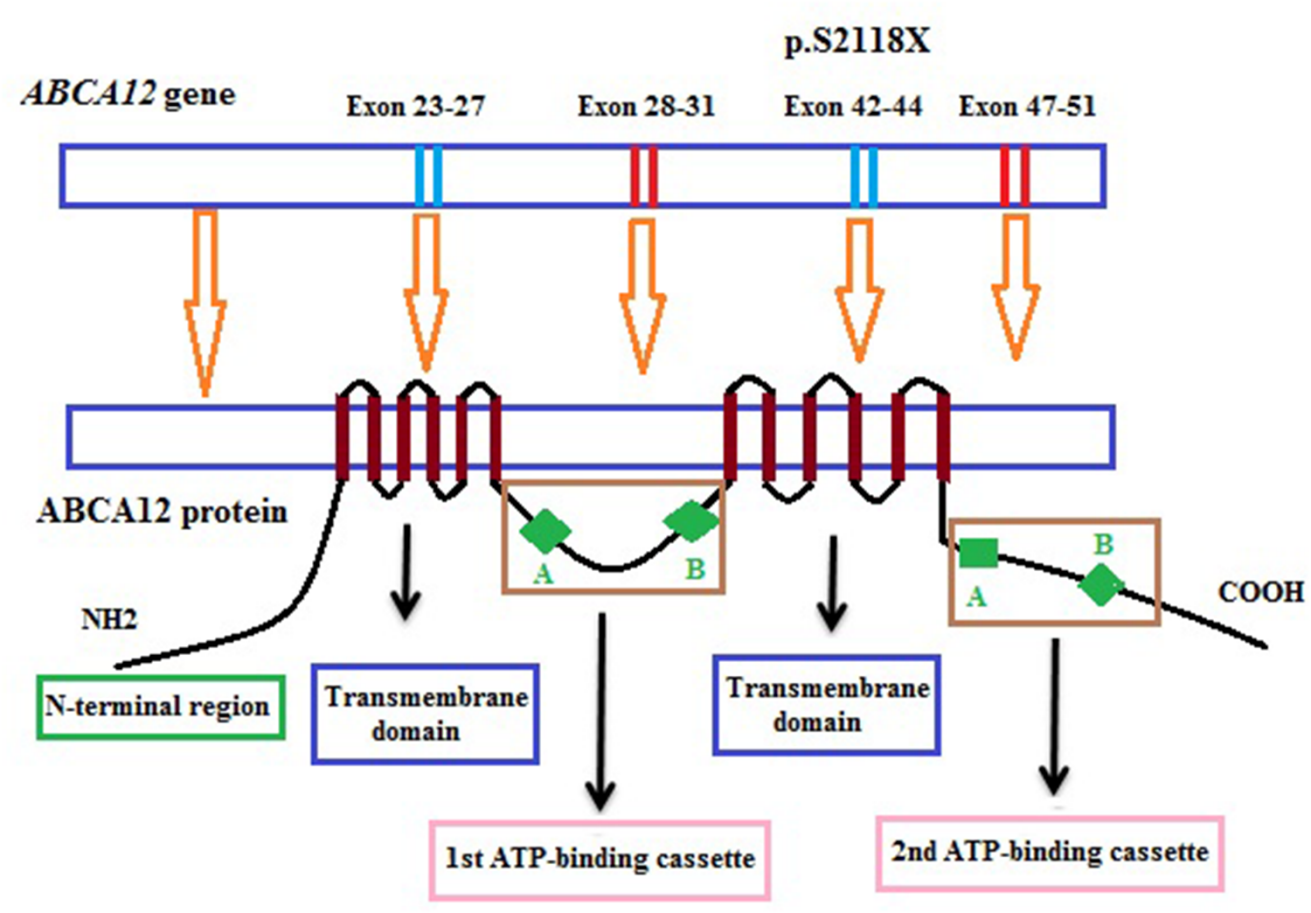
Position of the mutation in the structure model of the *ABCA12* gene and ABCA12 protein.

In our study, one novel homozygous mutation, c.6353C > G (*p*.S2118X) in the *ABCA12* gene, was identified in the patient by the WES sequencing method. This mutation results in the truncation ABCA12 protein that the loss of a part of the transmembrane domain and the total of the second ATP-binding cassette as well as the C-terminal region (Figure 3). According to the report of Yamanaka et al. (31), at least 62.5% of all the reported mutations are predicted to result in truncated proteins. In HI patients, most detected mutations are homozygous truncation mutations or compound with the heterozygous truncation mutations. Mortality is almost exclusively associated with homozygous mutations (3). The formed protein is severely dysfunctional and this is responsible for the serious phenotype in the patient. In addition, in this study, the heterozygous mutation was found in other clinically asymptomatic members of the patient's family including parents, one brother, and one sister. These results highlight the importance of genetic screening and prenatal diagnosis for families with a history of the disease.

In the cases of HI, prior to the identification of *ABCA12* as the causative gene, prenatal diagnoses were made by fetal skin biopsy and observation by electron microscopy in the later stages of pregnancies, with estimated gestational age in 19–23 weeks (32). The features of harlequin ichthyosis have been reported by ultrasound in the second trimester onward, such as floating particles in the amniotic fluid, enlarged anterior region of the eyeballs, characteristic face, and contracted and edema hands and feet (33). Therefore, prenatal diagnosis can be made by ultrasound. However, in some cases, due to the slow phenotypic development, ultrasound imaging may not be applicable (26). After the identification of *ABCA12* as the causative gene for HI, DNA-based prenatal diagnosis is possible at a much earlier stage of pregnancy. Genetic diagnosis has significantly reduced the risk to fetal health and reduced the burden on the family, especially in the family history of the disease or a carrier of serious genetic disease (34, 35). Especially in our patient's family with a history of giving birth to an infant with HI, genetic screening and DNA-based prenatal diagnosis are essential to delivering healthy babies.

## Conclusions

In our study, we performed WES analysis and identified a novel pathogenic mutation c.6353C > G (*p*.S2118X, Hom) in the *ABCA12* gene in a Vietnamese patient with HI. The result provide knowledge of the pathogenic mutation and shows that WES sequencing is an effect tool for detecting the novel mutation in Vietnamese patient with HI. The result of our study also emphasizes the need to diagnose carriers as well as assist in genetic counseling and prenatal screening in the HI patient's family.

## Data Availability

The datasets presented in this study can be found in online repositories. The data can be found here: BioProject ID: PRJNA921929 (https://www.ncbi.nlm.nih.gov/bioproject/921929) and SRA ID: SRR23019314 (https://www.ncbi.nlm.nih.gov/sra/?term=SRR23019314).

## References

[1] Akiyama M Sugiyama-Nakagiri Y Sakai K McMillan JR Goto M Arita K Mutations in lipid transporter ABCA12 in harlequin ichthyosis and functional recovery by corrective gene transfer. J Clin Invest. (2005) 115(7):1777–84. 10.1172/JCI24834 16007253 PMC1159149

[2] Thomas A Cullup T Norgett EE Kelsell DP. ABCA12 Is the major harlequin ichthyosis gene. J Invest Dermatol. (2006) 126(11):2408–13. 10.1038/sj.jid.5700455 16902423

[3] Rajpopat S Moss C Mellerio J Vahlquist A Ganemo A Hellstrom-Pigg M Harlequin ichthyosis: a review of clinical and molecular findings in 45 cases. Arch Dermatol. (2011) 147(6):681–6. 10.1001/archdermatol.2011.9 21339420

[4] Ahmed H O’Toole EA. Recent advances in the genetics and management of harlequin ichthyosis. Pediatr Dermatol. (2014) 31(5):539–46. 10.1111/pde.12383 24920541

[5] Shruthi B Nilgar BR Dalal A Limbani N. Harlequin ichthyosis: a rare case. Turk J Obstet Gynecol. (2017) 14(2):138–40. 10.4274/tjod.63004 28913151 PMC5558415

[6] Moskowitz DG Fowler AJ Heyman MB Cohen SP Crumrine D Elias PM Pathophysiologic basis for growth failure in children with ichthyosis: an evaluation of cutaneous ultrastructure, epidermal permeability barrier function, and energy expenditure. J Pediatr. (2004) 145(1):82–92. 10.1016/j.jpeds.2004.03.052 15238912

[7] Harvey HB Shaw MG Morrell DS. Perinatal management of harlequin ichthyosis: a case report and literature review. J Perinatol. (2010) 30:66–72. 10.1038/jp.2009.100 20038941

[8] Kelsell DP Norgett EE Unsworth H Teh MT Cullup T Mein CA Mutations in ABCA12 underlie the severe congenital skin disease harlequin ichthyosis. Am J Hum Genet. (2005) 76(5):794–803. 10.1086/429844 15756637 PMC1199369

[9] Annilo T Shulenin S Chen ZQ Arnould I Prades C Lemoine C Identification and characterization of a novel ABCA subfamily member, ABCA12, located in the lamellar ichthyosis region on 2q34. Cytogenet Genome Res. (2002) 98:169–76. 10.1159/000069811 12697999

[10] Lef´evre C Audebert S Jobard F Bouadjar B Lakhdar H Boughdene-Stambouli O Mutations in the transporter ABCA12 are associated with lamellar ichthyosis type 2. Hum Mol Genet. (2003) 12(18):2369–78. 10.1093/hmg/ddg235 12915478

[11] Borst P Elferink RO. Mammalian ABC transporters in health and disease. Annu Rev Biochem. (2002) 71:537–92. 10.1146/annurev.biochem.71.102301.093055 12045106

[12] Akiyama M. Harlequin ichthyosis and other autosomal recessive congenital ichthyoses: the underlying genetic defects and pathomechanisms. J Dermatol Sci. (2006) 42(2):83–9. 10.1016/j.jdermsci.2006.01.003 16481150

[13] Akiyama M. Updated molecular genetics and pathogenesis of ichthyoses. Nagoya J Med Sci. (2011a) 73:79–90. PMC4831217 21928690 PMC4831217

[14] Sakai K Akiyama M Sugiyama-Nakagiri Y McMillan JR Sawamura D Shimizu H. Localization of ABCA12 fromGolgi apparatus to lamellar granules in human upper epidermal keratinocytes. Exp Dermatol. (2007) 16(11):920–6. 10.1111/j.1600-0625.2007.00614.x 17927575

[15] Hovnanian A. Harlequin ichthyosis unmasked: a defect of lipid transport. J Clin Invest. (2005) 115(7):1708–10. 10.1172/JCI25736 16007249 PMC1159155

[16] Akiyama M Shimizu H. An update on molecular aspects of the non-syndromic ichthyoses. Exp Dermatol. (2008) 17(5):373–82. 10.1111/j.1600-0625.2007.00691.x 18341575

[17] Oji V Tadini G Akiyama M Bardon CB Bodemer C Bourrat E Revised nomenclature and classification of inherited ichthyoses: results of the first ichthyosis consensus conference in soreze 2009. J Am Acad Dermatol. (2010) 63(4):607–41. 10.1016/j.jaad.2009.11.020 20643494

[18] Liu J Zhang X Wang W Lan X Dong M Yan K Case report: prenatal diagnosis of a fetus with harlequin ichthyosis identifies novel compound heterozygous variants: a case report. Front Genet. (2021) 11:608196. 10.3389/fgene.2020.608196 33510771 PMC7835937

[19] Rubio-Gomez GA Weinstein M Pope E. Development of a disease severity score for newborns with collodion membrane. J Am Acad Dermatol. (2014) 70(3):506–11. 10.1016/j.jaad.2013.11.002 24373778

[20] Shibata A Akiyama M. Epidemiology, medical genetics, diagnosis and treatment of harlequin ichthyosis in Japan. Pediatr Int. (2015) 57(4):516–22. 10.1111/ped.12638 25857373

[21] Li H Durbin R. Fast and accurate short read alignment with Burrows-Wheeler transform. Bioinformatics. (2009) 25(14):1754–60. 10.1093/bioinformatics/btp324 19451168 PMC2705234

[22] Van der Auwera GA Carneiro MO Hartl C Poplin R del Angel G Levy-Moonshine A From FastQ data to high-confidence variant calls: the genome analysis toolkit best practices pipeline. Curr Protoc Bioinformatics. (2013) 43(1):11–0. 10.1002/0471250953.bi1110s43 PMC4243306 25431634

[23] Elias PM Fartasch M Crumrine D Behne M Uchida Y Holleran WM. Origin of the corneocyte lipid envelope (CLE): observations in harlequin ichthyosis and cultured human keratinocytes. J Invest Dermatol. (2000) 115(4):765–9. 10.1046/j.1523-1747.2000.00124-5.x 10998161

[24] Salehin S Azizimoghadam A Abdollahimohammad A Babaeipour-Divshali M. Harlequin ichthyosis: case report. J Res Med Sci. (2013) 18(11):1004–5. PMC3906774 24520234 PMC3906774

[25] Sheth JJ Bhavsar R Patel D Joshi A Sheth FJ. Harlequin ichthyosis due to novel splice site mutation in the *ABCA12* gene: postnatal toprenatal diagnosis. Int J Dermatol. (2018) 57(4):428–33. 10.1111/ijd.13923 29377090

[26] Hazuku T Yamada K Imaizumi M Ikebe T Shinoda K Nakatsuka K Unusual protrusion of conjunctiva in two neonates with harlequin ichthyosis. Case Rep Ophthalrnol. (2011) 2(1):73–7. 10.1159/000325138 PMC3072175 21475604

[27] Foca M Jakob K Whittier S Della Latta P Factor S Rubenstein D Endemic *Pseudomonas aeruginosa* infection in a neonatal intensive care unit. N Engl J Med. (2000) 343(10):695–700. 10.1056/NEJM200009073431004 10974133

[28] Parikh K Brar K Glick JB Flamm A Glick SA. A case report of fatal harlequin ichthyosis: insights into infectious and respiratory complications. JAAD Case Rep. (2016) 2(4):301–3. 10.1016/j.jdcr.2016.06.011 27536717 PMC4976614

[29] Akiyama M. *ABCA12* Mutations and autosomal recessive congenital ichthyosis: a review of genotype/phenotype correlations and of pathogenic concepts. Hum Mutat. (2010) 31(10):1090–6. 10.1002/humu.21326 20672373

[30] Akiyama M. The role of ABCA12 in keratinocyte differentiation and lipid barrier formation in the epidermis. Dermatoendocrinol. (2011b) 3(2):107–12. 10.4161/derm.3.2.15136 21695020 PMC3117010

[31] Yamanaka Y Akiyama M Sugiyama-Nakagiri Y Sakai K Goto M McMillan JR Expression of the keratinocyte lipid transporter ABCA12 in developing and reconstituted human epidermis. Am J Pathol. (2007) 171(1):43–52. 10.2353/ajpath.2007.061207 17591952 PMC1941601

[32] Shimizu A Akiyama M Ishiko A Yoshiike T Suzumori K Shimizu H. Prenatal exclusion of harlequin ichthyosis; potential pitfalls in the timing of the fetal skin biopsy. Br J Dermatol. (2005) 153(4):811–4. 10.1111/j.1365-2133.2005.06778.x 16181466

[33] Zhou Y Zhuang DZ Han R Isaac G Tobin JJ McKee M ABCA12 Maintains the epidermal lipid permeability barrier by facilitating formation of ceramide linoleic esters. J Biol Chem. (2021) 283(52):36624–35. 10.1074/jbc.M807377200 PMC2605993 18957418

[34] Akiyama M Titeux M Sakai K McMillan JR Tonasso L Calvas P DNA-based prenatal diagnosis of harlequin ichthyosis and characterization of ABCA12 mutation consequences. J Invest Dermatol. (2007) 127(3):568–73. 10.1038/sj.jid.5700617 17082782

[35] Yanagi T Akiyama M Sakai K Nagasaki A Ozawa N Kosaki R DNA-based prenatal exclusion of harlequin ichthyosis. J Am Acad Dermatol. (2008) 58(4):653–6. 10.1016/j.jaad.2007.12.018 18262308

